# Consistently low levels of histidine-rich glycoprotein as a new prognostic biomarker for sepsis: A multicenter prospective observational study

**DOI:** 10.1371/journal.pone.0283426

**Published:** 2023-03-29

**Authors:** Naoya Kawanoue, Kosuke Kuroda, Hiroko Yasuda, Masahiko Oiwa, Satoshi Suzuki, Hidenori Wake, Hiroki Hosoi, Masahiro Nishibori, Hiroshi Morimatsu, on behalf of the Okayama Research Investigation Organizing Network (ORION)

**Affiliations:** 1 Department of Anesthesiology and Resuscitology, Okayama University Graduate School of Medicine, Dentistry and Pharmaceutical Sciences, Okayama, Japan; 2 Department of Pharmacology, Okayama University Graduate School of Medicine, Dentistry and Pharmaceutical Sciences, Okayama, Japan; 3 Data Science Division, Center for Innovative Clinical Medicine, Okayama University Hospital, Okayama, Japan; The University of Lahore, PAKISTAN

## Abstract

**Background:**

Few sepsis biomarkers accurately predict severity and mortality. Previously, we had reported that first-day histidine-rich glycoprotein (HRG) levels were significantly lower in patients with sepsis and were associated with mortality. Since the time trends of HRG are unknown, this study focused on the time course of HRG in patients with sepsis and evaluated the differences between survivors and non-survivors.

**Methods:**

A multicenter prospective observational study was conducted involving 200 patients with sepsis in 16 Japanese hospitals. Blood samples were collected on days 1, 3, 5, and 7, and 28-day mortality was used for survival analysis. Plasma HRG levels were determined using a modified quantitative sandwich enzyme-linked immunosorbent assay.

**Results:**

First-day HRG levels in non-survivors were significantly lower than those in survivors (mean, 15.7 [95% confidence interval (CI), 13.4–18.1] vs 20.7 [19.5–21.9] μg/mL; *P* = 0.006). Although there was no time × survivors/non-survivors interaction in the time courses of HRG (*P* = 0.34), the main effect of generalized linear mixed models was significant (*P* < 0.001). In a univariate Cox proportional hazards model with each variable as a time-dependent covariate, higher HRG levels were significantly associated with a lower risk of mortality (hazard ratio, 0.85 [95% CI, 0.78–0.92]; *P* < 0.001). Furthermore, presepsin levels (*P* = 0.02) and Sequential Organ Function Assessment scores (*P* < 0.001) were significantly associated with mortality. Harrell’s C-index values for the 28-day mortality effect of HRG, presepsin, procalcitonin, and C-reactive protein were 0.72, 0.70, 0.63, and 0.59, respectively.

**Conclusions:**

HRG levels in non-survivors were consistently lower than those in survivors during the first seven days of sepsis. Repeatedly measured HRG levels were significantly associated with mortality. Furthermore, the predictive power of HRG for mortality may be superior to that of other singular biomarkers, including presepsin, procalcitonin, and C-reactive protein.

## Introduction

Sepsis is a life-threatening organ dysfunction caused by a dysregulated host response to infection [1]. Globally, approximately 48.9 million patients suffer from sepsis annually, and 11.0 million (19.7%) of all deaths are sepsis-related [2]. Although the sepsis mortality rate has been decreasing every year owing to efforts in early diagnosis and treatment [2–4], sepsis remains a leading cause of death and a major public health concern [2, 3]. The clinical course of sepsis is unpredictable, and the current situation has caused serious problems in predicting whether a patient will deteriorate or recover soon. To overcome this issue, better indicators of severity in patients with sepsis are needed. The first-day value and time-dependent changes in the Sequential Organ Function Assessment (SOFA) score are useful prognostic markers [1, 5–8], however, the usefulness of presepsin (P-SEP), procalcitonin (PCT), and C-reactive protein (CRP) levels is controversial [7–10].

Histidine-rich glycoprotein (HRG) is a 75 kDa plasma glycoprotein that is mainly produced in the liver [11]; it is present at a concentration of approximately 60–150 μg/mL in healthy individuals [12, 13]. HRG binds to a broad range of ligands and is implicated in regulating coagulation, fibrinolysis, and immune response [13–15]. We chose HRG as a possible biomarker for sepsis because we assumed that decreased plasma HRG levels in sepsis would cause the dysregulation of coagulation, fibrinolysis, and immune response, resulting in disseminated intravascular coagulation and multiple organ dysfunction syndrome [14, 16–18]. Previously, using a cecal ligation and puncture model, we showed that HRG levels decreased in septic mice and that replenishment of HRG improved their survival rate [18]. In our clinical study, we reported that first-day HRG levels were lower in patients with sepsis than in those without sepsis [12]; moreover, lower HRG levels were associated with an increased risk of septic mortality [19], suggesting that first-day HRG levels might be a biomarker for sepsis [12, 16, 17, 19]. However, owing to a limited sample size and lack of knowledge on how HRG levels change, the prognostic value of HRG in sepsis was not fully evaluated. Herein, for the first time, we aimed to determine the trends of plasma HRG levels in patients with sepsis and evaluate the differences between survivors and non-survivors.

## Materials and methods

### Study design

A multicenter prospective observational study was conducted with the Institutional Review Board approval of all relevant institutions: the Okayama University Graduate School of Medicine, Dentistry, and Pharmaceutical Sciences (Ethical Number: 1801–020), Japanese Red Cross Okayama Hospital (R1-33), Okayama saiseikai General Hospital (180201), Onomichi Municipal Hospital (19–11), Kagawa Prefectural Central Hospital (786), National Hospital Organization Fukuyama Medical Center (H29-40), Tsuyama Chuo Hospital (421), Shimane University Hospital (3999), Japanese Red Cross Society Himeji Hospital (2019–30), Kagawa University Hospital (H30-006), Tottori University Hospital (18A014), Fukuyama City Hospital (365), Kawasaki Medical School General Medical Center (3006–2), Japanese Red Cross Kobe Hospital (154), Okayama City Hospital (1–116), and Kawasaki Medical School Hospital (3265). This study was registered with the UMIN Clinical Trials Registry on February 1, 2018 (UMIN000030037; the lead principal investigator is Hiroshi Morimatsu). This study was performed in accordance with the World Medical Association Declaration of Helsinki: ethical principles for medical research involving human subjects. This observational study was based on the Strengthening the Reporting of Observational Studies in Epidemiology guidelines [20].

### Patients and data collection

Patients newly diagnosed with sepsis based on the Sepsis-3 definition [1] were prospectively enrolled in the study. SOFA scores were calculated from PaO_2_/F_I_O_2_ ratio, use of mechanical ventilation, platelet count, total bilirubin, blood pressure, used inotropes and vasopressors, Glasgow Coma Scale, creatinine, and urine output [6]. The inclusion criteria included admission to the intensive care unit (ICU) with an increase in the SOFA score by 2 points or more caused by a dysregulated host response to infection. The exclusion criteria were as follows: age less than 20 years, pregnancy, and failure to provide written consent. After obtaining written consent from patients or their relatives, blood samples were collected to be analyzed later for HRG, P-SEP, and PCT levels on days 1 (within 24 h of diagnosis of sepsis), 3, 5, and 7, while the patients stayed in the ICU. Daily clinical and blood sampling data were also recorded using the Research Electronic Data Capture system in each hospital, and the Acute Physiology and Chronic Health Evaluation (APACHE) II score on day 1 [21] and SOFA scores on days 1, 3, 5, and 7 were calculated. The patients were then observed for 90 days through medical records and telephone follow-up in each hospital, and the 28-day mortality values were used for survival analysis.

### Measurement methods

Blood samples were collected in tubes containing dipotassium-ethylenediaminetetraacetic acid (EDTA) and processed within 60 min of sampling. Samples were centrifuged at 3,000 rpm for 10 min, and the plasma was collected in polypropylene tubes. The samples were immediately frozen at each hospital. Thereafter, the samples were transported from each hospital to Okayama University, and a protease inhibitor cocktail (cOmplete, Mini, EDTA-free; Roche Diagnostics, Basel, Switzerland) was added to the samples according to the manufacturer’s instructions prior to their storage at –80°C.

Plasma HRG levels were determined using a previously reported in-house modified quantitative sandwich enzyme-linked immunosorbent assay in S3 Fig [12]. Briefly, a rat monoclonal antibody against human HRG (in-house, #75–14) was used as the capture antibody, and a nitrilotriacetate nickel (Ni^2+^)-activated derivative of horseradish peroxidase (HisProbe-HRP Conjugate; Thermo Fisher Scientific, Waltham, MA) diluted by 1000-fold was used for detection. Plasma samples were diluted by 200- and 400-fold with phosphate-buffered saline containing 1% bovine serum albumin. A 96-well plate (Clear Flat-Bottom Immuno Nonsterile 96-Well Plates; Thermo Fisher Scientific) and a microplate washer (ImmunoWash 1575 Microplate Washer; Bio-Rad Laboratories, Hercules, CA) were used. O-phenylenediamine (SIGMAFAST OPD tablet; Sigma-Aldrich, St. Louis, MO) and stop solutions (3 M H_2_SO_4_) were used to develop the reaction. The absorbance was measured at 492 nm using a 96-well plate reader (Nivo 5S Multimode Plate Reader; PerkinElmer, Waltham, MA), and a standard curve was generated using serial dilutions of a known amount of purified HRG (manufactured in-house). Plasma samples were measured in duplicate, and HRG levels were determined by averaging two independent assays. P-SEP and PCT levels were determined using a chemiluminescent enzyme immunoassay (SRL, Tokyo, Japan).

### Outcomes

The primary outcome of this study was all-cause mortality within 28 days of the initial sampling. The differences in the time courses of HRG levels between survivors and non-survivors were evaluated. The secondary outcome was the association between each variable (HRG, P-SEP, PCT, CRP, SOFA score, and APACHE II score) and mortality.

### Statistical analysis

All the statistical methods were designed a priori, except for the statistical analysis shown in S1 Fig. This subgroup analysis was designed as a post-hoc analysis because it was conducted while exploring the data.

Continuous variables are presented as the median (interquartile range [IQR], 25th to 75th percentiles) or the mean (95% confidence interval [CI]), and differences between survivors and non-survivors were analyzed using the Mann–Whitney U test. Categorical variables are presented as proportions, and differences between survivors and non-survivors were analyzed using Fisher’s exact test. The time-dependent changes in each variable in survivors and non-survivors were compared using generalized linear mixed models (GLMMs) for repeated measures. The Cox proportional hazards model and Kaplan–Meier method were used for survival analysis. The ability of each variable to predict 28-day mortality was evaluated using the Cox proportional hazards model with each variable as a time-dependent covariate and then adjusted for the APACHE II score to correct for the severity of disease. The hazard ratios (HRs) and 95% CIs were estimated. In addition, patients with sepsis were stratified according to background factors to evaluate the association between HRG levels and 28-day mortality. After dividing patients with sepsis into two groups according to the cutoff value, which was derived from receiver operating characteristic analysis in logistic regression and determined using the Youden index method, cumulative survival rates were estimated using the Kaplan–Meier method and tested using the log-rank test. The significance level of testing was set at 0.05 (two-sided *p* value). IBM SPSS Statistics (version 25.0; International Business Machines Corporation, Armonk, NY) was used to analyze the GLMM, Stata 17.0 (StataCorp LLC, College Station, TX) was used to analyze the Cox proportional hazards model with time-dependent covariates, and JMP Pro 14.0.0 (SAS Institute Inc., Cary, NC) was used for the other analyses.

## Results

### Patient characteristics

**Fig 1:**
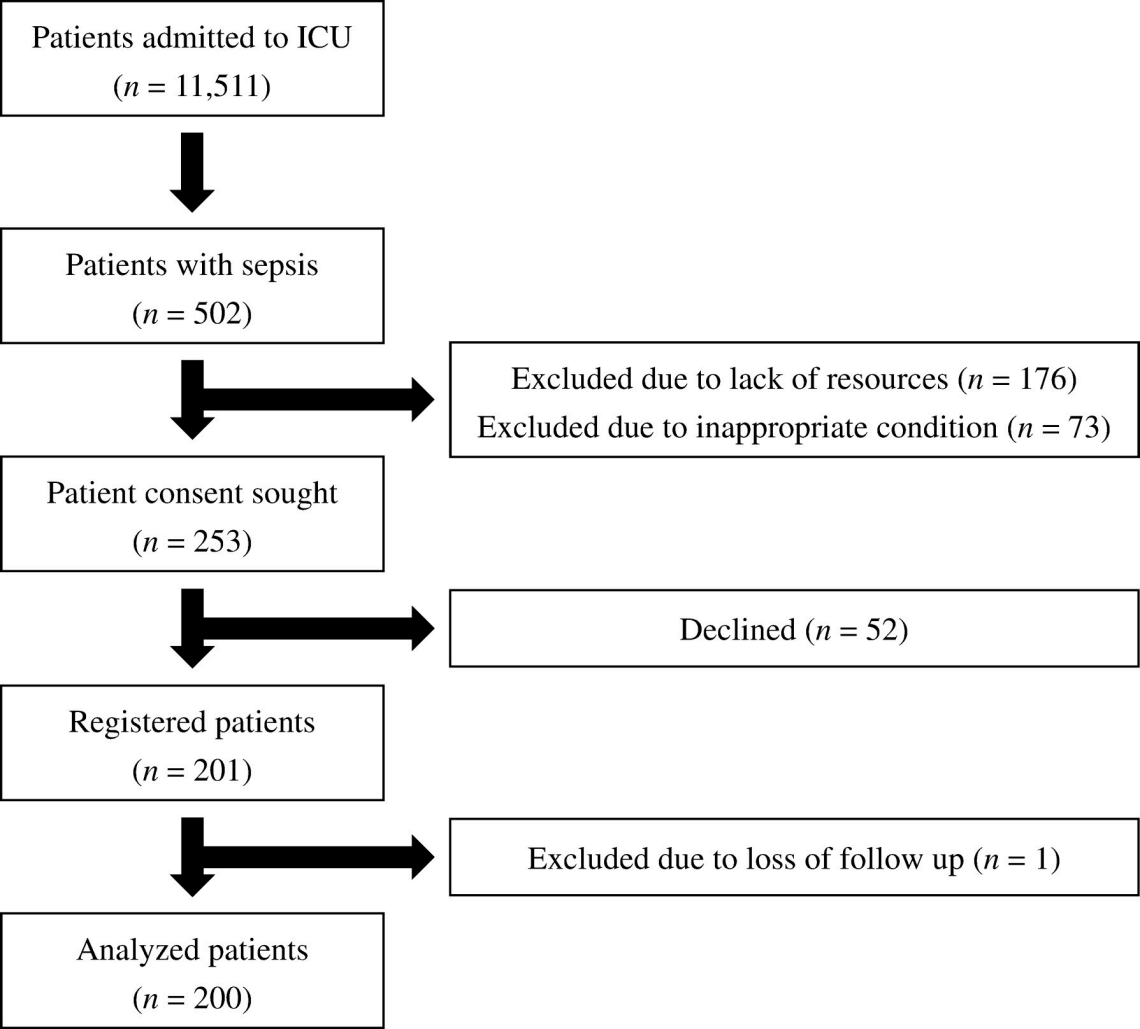
Patient flow chart.

Patients were registered from August 2018 to September 2019 in 16 Japanese hospitals: 5 university and 11 general hospitals. During the study, 11,511 patients were admitted to the ICU and 502 patients (4.4%) were diagnosed with sepsis. Written informed consent was obtained from 201 patients, and one patient who failed to follow-up was excluded. Finally, 200 patients were analyzed in this study (Fig 1). Patient characteristics are shown in Table 1 and S1 Table. The median first-day APACHE II and SOFA scores were 25 (IQR, 20–31) and 10 (IQR, 7–12), respectively, and 132 patients (66.0%) were diagnosed with septic shock. The 28- and 90-day mortality rates were 11.5% (23 patients) and 19.0% (38 patients), respectively. The main causes of 28-day mortality were multiple organ dysfunction syndrome associated with exacerbation of the primary disease (18 cases), fatal arrhythmia (two cases), and others (three cases).

**Table 1:** Patient characteristics in survivors and non-survivors.

Variables	Total (*n* = 200)	Survivors (*n* = 177)	Non-survivors (*n* = 23)	*P* value
Age, median (IQR), years	75 (67–83)	75 (67–83)	75 (67–86)	0.73
Sex, male	110 (55.0%)	94 (53.1%)	16 (69.6%)	0.18
Height, median (IQR), cm	160 (150–165)	160 (150–166)	158 (150–164)	0.74
Weight, median (IQR), kg	55 (46–65)	55 (47–65)	49 (46–59)	0.13
Surgical admission	61 (30.5%)	57 (32.2%)	4(17.4%)	0.23
APACHE II score*, median (IQR)	25 (20–31)	25 (19–30)	35 (24–41)	< 0.001
SOFA score*, median (IQR)	10 (7–12)	10 (7–12)	13 (11–16)	< 0.001
Septic shock	132 (66.0%)	112 (63.3%)	20 (87.0%)	0.03
Inotropes/vasopressors	160 (80.0%)	139 (78.5%)	21 (91.3%)	0.18
Acute kidney injury	109 (54.5%)	93 (52.5%)	16 (69.6%)	0.18
Renal replacement therapy	33 (16.5%)	24 (13.6%)	9 (39.1%)	0.005
Mechanical ventilation	99 (49.5%)	83 (46.9%)	16 (69.6%)	0.047
Source of infection				0.10
Lung	51 (25.5%)	39 (22.0%)	12 (52.2%)	
Urinary tract	38 (19.0%)	36 (20.3%)	2 (8.7%)	
Gastrointestinal tract	36 (18.0%)	33 (18.6%)	3 (13.0%)	
Hepatobiliary tract	26 (13.0%)	25 (14.1%)	1 (4.3%)	
Bone and soft tissue	20 (10.0%)	18 (10.2%)	2 (8.7%)	
Others	29 (14.5%)	26 (14.7%)	3 (13.0%)	

* On day 1

### Time course of plasma HRG levels and other markers

S2 Table shows mean HRG levels on days 1, 3, 5, 7, and last observation till day 7 carried forward (LOCF) in survivors and non-survivors. HRG level in non-survivors was significantly lower than that in survivors on each day (*P* = 0.006, 0.001, 0.004, 0.02, and < 0.001, respectively).

**Fig 2:**
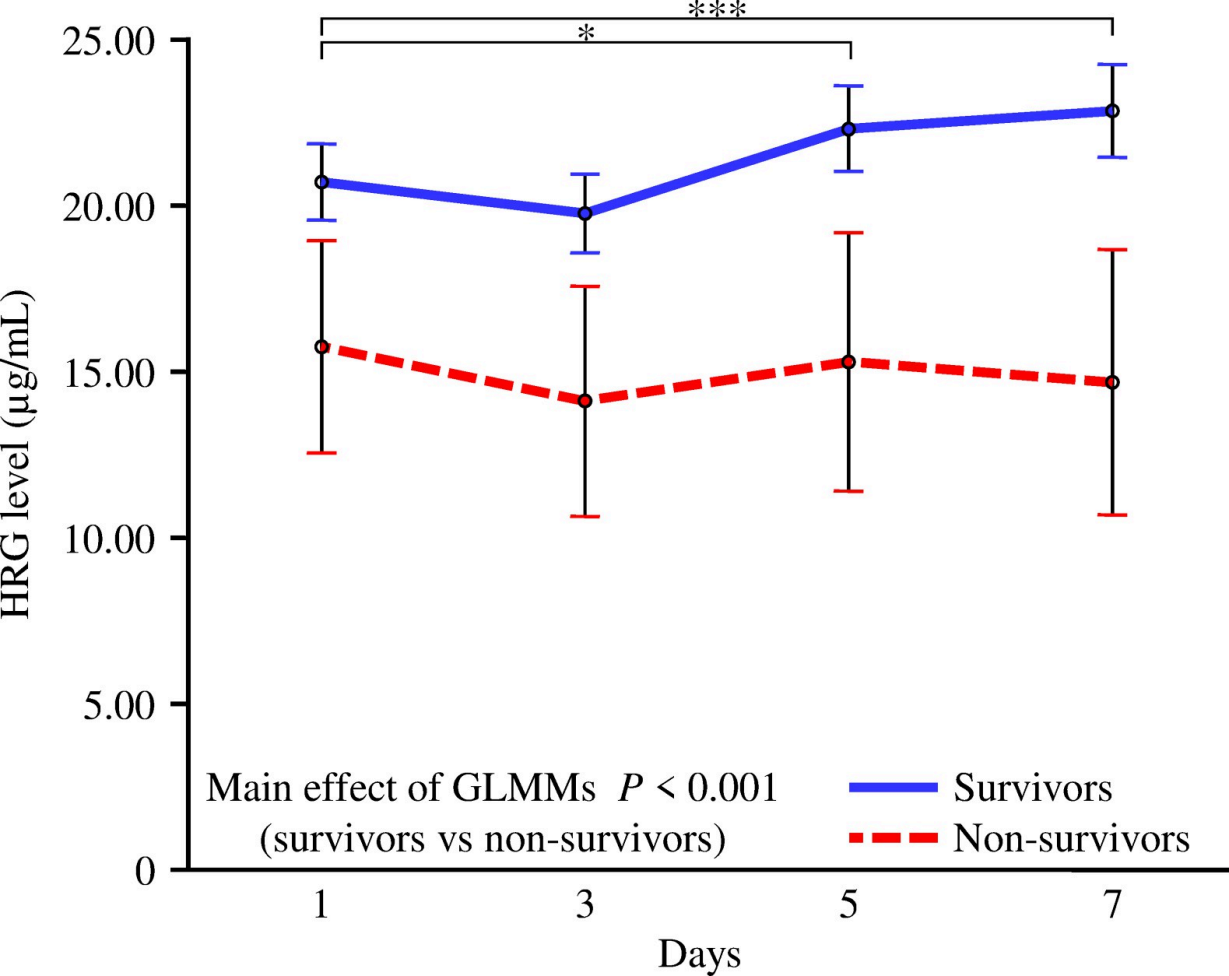
Comparative time courses of plasma HRG levels in survivors and non-survivors. GLMMs were used to compare the time-dependent changes in plasma HRG levels between survivors (*n* = 177) and non-survivors (*n* = 23) among patients with sepsis. There was no time × survivors/non-survivors interaction in the time-dependent changes in HRG (*P* = 0.34). However, the main effect of GLMMs was significant (*P* < 0.001), and HRG levels in non-survivors remained consistently lower than those in survivors. Contrast test in GLMMs; *P = 0.004, ***P < 0.001.

Fig 2 shows the comparative time courses of HRG in survivors and non-survivors obtained using GLMMs. Although the HRG was unchanged at low levels up to 7 days in non-survivors and recovered from day 5 in survivors, there was no time × survivors/non-survivors interaction in their time-dependent changes (*P* = 0.34). However, the main effect of GLMMs was significant (*P* < 0.001), and HRG levels in non-survivors remained consistently lower than those in survivors.

**Fig 3:**
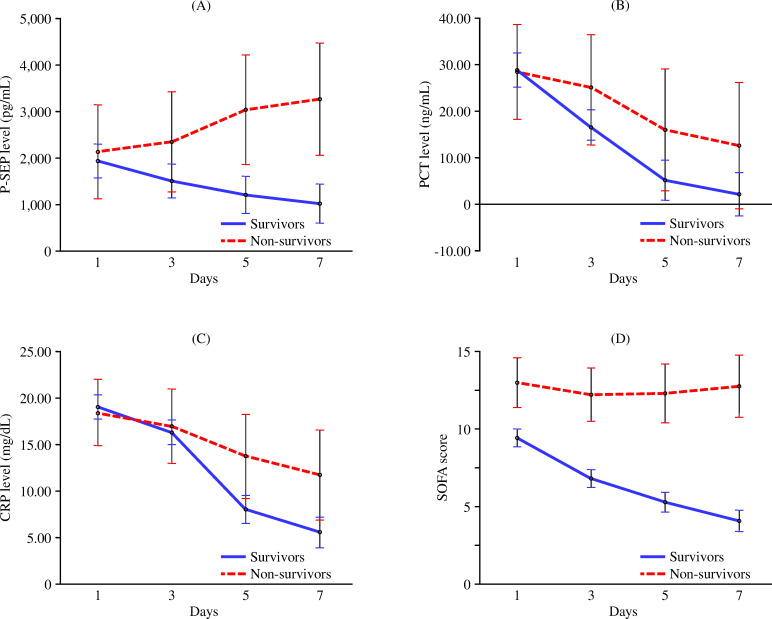
Comparative time courses of each variable in survivors and non-survivors. GLMMs were used to compare the time-dependent changes in each variable, including (A) P-SEP, (B) PCT, and (C) CRP levels, as well as (D) SOFA scores, between survivors (*n* = 177) and non-survivors (*n* = 23) among patients with sepsis. (A) There was a significant time × survivors/non-survivors interaction in the time-dependent changes in P-SEP (*P* < 0.001). The main effect of GLMMs was significant (*P* = 0.02), and P-SEP levels in non-survivors were higher than those in survivors. (B) There was no significant time × survivors/non-survivors interaction in the time-dependent changes in PCT (*P* = 0.23). The main effect of GLMMs was not significant (*P* = 0.14), and PCT levels in survivors and non-survivors were not different. (C) There was a significant time × survivors/non-survivors interaction in the time-dependent changes in CRP (*P* = 0.01). However, the main effect of GLMMs was not significant (*P* = 0.08), and CRP levels in survivors and non-survivors were not different. (D) There was a significant time × survivors/non-survivors interaction in the time-dependent changes in SOFA scores (*P* < 0.001). The main effect of GLMMs was significant (*P* < 0.001), and SOFA scores in non-survivors remained consistently higher than those in survivors.

Fig 3 shows differences in time courses of P-SEP, PCT, CRP, and SOFA scores between survivors and non-survivors using GLMMs. There was a significant time × survivors/non-survivors interaction in the time-dependent changes in P-SEP (*P* < 0.001), with an increase in non-survivors and a decrease in survivors up to seven days; the main effect of GLMMs was also significant (*P* = 0.02). There was no significant time × survivors/non-survivors interaction in the time-dependent changes in PCT (*P* = 0.23), and the main effect of GLMMs was not significant (*P* = 0.14). There was a significant time × survivors/non-survivors interaction in the time-dependent changes in CRP (*P* = 0.01), but the main effect of GLMMs was not significant (*P* = 0.08). There was a significant time × survivors/non-survivors interaction in the time-dependent changes in SOFA scores (*P* < 0.001), which remained unchanged in non-survivors and decreased in survivors over time. The main effect of GLMMs was also significant (*P* < 0.001), and SOFA scores in non-survivors remained consistently higher than those in survivors.

### Association between markers and mortality

Table 2 shows the association between each variable and the 28-day mortality. In the univariate Cox proportional hazards model with each variable as a time-dependent covariate, higher HRG levels were significantly associated with a lower risk of mortality (HR, 0.85 [95% CI, 0.78–0.92]; *P* < 0.001). The Harrell’s C-index value of HRG for mortality was 0.72. P-SEP (HR, 1.01 [95% CI, 1.002–1.02]; *P* = 0.02) and SOFA scores (HR, 1.34 [95% CI, 1.24–1.45]; *P* < 0.001) were also significantly associated with a risk of mortality. However, PCT and CRP were not associated with mortality (*P* = 0.20 and 0.34, respectively). After adjusting for the first-day APACHE II score, HRG levels (adjusted HR, 0.88 [95% CI, 0.81–0.96]; *P* = 0.003) and SOFA scores (adjusted HR, 1.34 [95% CI, 1.19–1.51]; *P* < 0.001) remained significant prognostic factors.

**Table 2:** Association between each variable and 28-day mortality.

	Univariate analysis			Adjusted for APACHE II score*	
Variables	HR (95% CI)	*P* value	Harrell’s C-Index	Adjusted HR (95% CI)	*P* value
HRG	0.85 (0.78–0.92)	< 0.001	0.72	0.88 (0.81–0.96)	0.003
P-SEP (/100)	1.01 (1.002–1.02)	0.02	0.70	1.00 (0.99–1.02)	0.58
PCT	1.01 (0.99–1.03)	0.20	0.63	1.00 (0.99–1.02)	0.74
CRP	1.02 (0.98–1.07)	0.34	0.59	1.03 (0.99–1.08)	0.13
SOFA score	1.34 (1.24–1.45)	< 0.001	0.87	1.34 (1.19–1.51)	< 0.001
APACHE II score*	1.12 (1.07–1.17)	< 0.001	0.74		
Lactate*	1.15 (1.06–1.25)	0.001	0.71		

* On day 1

The Cox proportional hazards model with each variable as a time-dependent covariate was used to evaluate associations between each variable and 28-day mortality. APACHE II score and lactate were only used for first-day values. Adjusted HR denotes the HR adjusted for first-day APACHE II score.

The sensitivity, specificity, and positive and negative predictive values of the first-day HRG levels associated with mortality at the cutoff level of 14.03 μg/mL were 0.48, 0.83, 0.27, and 0.92, respectively. When patients with sepsis were divided into high- and low-HRG groups according to this cutoff level, the Kaplan–Meier survival curves (S2 Fig) showed that mortality in the low-HRG group was significantly higher than that in the high-HRG group (log-rank test, *P* < 0.001).

### Subgroup analysis

S1 Fig shows the association between HRG levels and 28-day mortality in subgroups stratified by the following background factors: APACHE II score, septic shock, creatinine, bilirubin, and lung infection. In the univariate Cox proportional hazards model with time-dependent covariates, higher HRG levels were significantly associated with a lower risk of mortality in all subgroups, except in the group with bilirubin levels of 2 mg/dL or higher.

## Discussion

We demonstrated that first-day HRG levels in non-survivor patients with sepsis were significantly lower than those in survivors and remained consistently lower during the first seven days in the ICU. Repeatedly measured HRG levels were significantly associated with mortality in the acute phase of sepsis. These characteristics of HRG were confirmed in patients with varying severity, organ damage, and source of infection.

Previously, we revealed that plasma HRG levels were markedly decreased due to a rapid reduction in HRG mRNA expression in the liver, deposition of HRG on immunothrombi, and degradation of HRG by thrombin in a sepsis state [18]. And we reported that first-day HRG levels in patients with sepsis (*n* = 20) were significantly lower than those in patients with non-infective systemic inflammatory response syndrome (*n* = 50) in a single-center [12]; HRG levels in non-survivors (*n* = 16) were significantly lower than those in survivors (*n* = 83) among patients with sepsis in 11 Japanese hospitals [19]. Consistent with our previous studies, first-day HRG levels in non-survivors were significantly lower than those in survivors in this study. Regarding the time-dependent change in HRG, survivors showed recovery from day 5, and HRG levels on days 5 and 7 were significantly higher than those on day 1. In contrast, HRG levels in non-survivors remained low and unchanged. HRG levels in non-survivors were consistently lower than those in survivors during the observational period. These characteristics were similar to those observed in the SOFA scores. Regarding other markers, first-day P-SEP levels were similar between survivors and non-survivors, but there was a significant difference in its time-dependent changes, consistent with previous reports [8]. In the present study, PCT and CRP levels decreased similarly over time in both survivors and non-survivors as in previous studies [8, 9]. We showed that the repeatedly measured HRG, P-SEP, and SOFA scores, and not PCT and CRP, were significantly associated with mortality in the acute phase of sepsis. Although several studies have reported the usefulness of P-SEP as a prognostic biomarker for sepsis, similar to the present study, it is difficult to predict the prognosis of sepsis based on PCT and CRP levels [7–9]. SOFA and APACHE II scores require the calculation of multiple items; these scores are well-established prognostic indicators for critically ill patients in the ICU, especially patients with severe sepsis [1, 5, 6]. The ability of HRG to predict mortality was inferior to that of the SOFA score but superior to that of other single biomarkers, including P-SEP, PCT, and CRP. To confirm the reliability of HRG as a prognostic biomarker, we performed additional analyses, such as APACHE II-adjusted and subgroup analyses, which demonstrated that HRG levels were efficient prognostic biomarkers in the acute phase of sepsis.

We demonstrated that HRG levels in non-survivor patients with sepsis were always different from those in survivors throughout the first 7 days in the ICU. This trend was significant only for HRG levels and SOFA scores. The initial levels of these two markers were different in survivors and non-survivors; they improved in survivors but not in non-survivors. These findings are important for assessing patient responses to treatment in clinical settings. Levels of other biomarkers, including P-SEP, PCT, and CRP, showed a little difference between survivors and non-survivors on day 1, followed by a gradual increase in differences between the groups. Moreover, in a recent proteome study on patients with severe coronavirus disease 2019, the initial HRG level was one of the most useful prognostic factors, and HRG levels in non-survivors were consistently lower than those in survivors at multiple subsequent measurements [22]. These results indicate that, in addition to the markers used clinically, HRG provides more reliable information about the clinical course in the acute phase of sepsis and would enable us to discriminate more severely patients who truly need multidisciplinary treatment. Furthermore, in our previous animal study, we reported that replenishment of HRG in septic mice with reduced HRG levels improved their survival rate [18], which might also provide a novel therapeutic strategy for supplementation of HRG in the future treatment of sepsis.

This study has some limitations. First, we enrolled 200 patients with sepsis from 16 hospitals, and the 28-day mortality, median first-day APACHE II score, and SOFA score were 11.5%, 25, and 10, respectively. The mortality rate was lower than expected. In Japan, in severe sepsis and septic shock based on the Sepsis-2 definition [23], the 28-day mortality, median APACHE II score, and SOFA score were reported to be 18.9%, 21–25, and 8–9, respectively, in 59 ICUs [24], and 18.0%, 24–28, and 9–13, respectively, in 11 ICUs [19]. However, we believe that our patient group was appropriate, considering the severity of the disease based on the APACHE II and SOFA scores. To confirm the capability of HRG as a prognostic biomarker for sepsis, it is advisable to conduct a larger study. Second, we collected limited data after ICU discharge and, therefore, lack data on the recovery period for survivors who were discharged early and data on non-survivors who died early. To compensate for missing data, we compared the time-dependent trends between survivors and non-survivors using GLMMs for repeated measures. The data reported in this study may have missed higher HRG levels in recovered survivors and lower HRG levels in non-survivors; hence, the premise of “missing at random” in GLMMs is unsatisfactory and the mean HRG difference between survivors and non-survivors might be biased. Because it was considered that the missing data might have led to an underestimation of the difference in HRG between survivors and non-survivors in GLMMs, LOCF analysis was performed to verify this issue. It was observed that the data were not underestimated because the mean day 7 HRG difference in GLMMs and LOCF was 8.18 and 7.47 μg/mL, respectively. Third, we did not have detailed data on confounding factors that affect HRG levels, such as age, liver function, malignancy status, and steroid use [25–28]. Therefore, we performed additional subgroup analyses to confirm the reliability of the HRG trends as a prognostic biomarker. However, the influence of these factors cannot be completely excluded and hence might have affected our data. Fourth, we revealed that HRG levels in non-survivor patients with sepsis were consistently lower than those in survivors. However, the mechanism of time trends in HRG after treatment of sepsis is not clear, therefore, further studies on HRG would be needed.

## Conclusions

HRG levels in non-survivor patients with sepsis were significantly lower than those in survivors on day 1 and remained consistently lower than those in survivors during the first 7 days in the ICU. Repeatedly measured HRG was significantly associated with mortality. We suggest that HRG is a useful diagnostic and prognostic biomarker in the acute phase of sepsis. However, large validation studies are needed to confirm our findings.

## Supporting information

S1 Table Patient characteristics in survivors and non-survivors. (PDF)

S2 Table Plasma HRG levels on days 1, 3, 5, 7, and LOCF in survivors and non-survivors. (PDF)

S1 Fig Association between plasma HRG levels and mortality in subgroups. The Cox proportional hazards model with time-dependent covariates was used to evaluate associations between HRG levels and 28-day mortality. Higher HRG levels were significantly associated with a lower risk of mortality in all subgroups, except in the group with bilirubin levels ≥ 2 mg/dL.
(PDF)

S2 Fig Kaplan–Meier survival curves. Patients with sepsis were divided into high- and low-HRG groups according to the cutoff level of 14.04 μg/mL. The sensitivity and specificity of first-day HRG levels associated with mortality were 0.48 and 0.83, respectively, at this cutoff level.
(PDF)

S3 Fig Schematic diagram of enzyme-linked immunosorbent assay. (PDF)

S1 Data (DOCX)
