# Supplementary material for: Consistently low levels of histidine-rich glycoprotein as a new prognostic biomarker for sepsis: A multicenter prospective observational study
Source: PLoS One. 2023 Mar 29;18(3):e0283426. doi: 10.1371/journal.pone.0283426 (PMC10057827; doi:10.1371/journal.pone.0283426)
Supplement: S1 Table — (PDF) [file pone.0283426.s001.pdf]

S1 Table. Patient characteristics in survivors and non-survivors (first-day blood sampling data) [median (IQR)].

Variables	Total (*n* = 200)	Survivors (*n* = 177)	Non-survivors (*n* = 23)	*P* value
HRG (μg/mL)	18.7 (14.8–23.7)	18.9 (15.0–24.5)	15.0 (11.2–19.9)	0.006
P-SEP (pg/mL)	1,065 (532–2,143)	992 (507–1,950)	1,570 (767–3,010)	0.03
PCT (ng/mL)	14.3 (3.4–49.8)	14.3 (3.2–49.5)	14.1 (3.4–58.1)	0.89
WBC (/L)	15,150 (9,473–21,630)	15,300 (9,600–21,450)	12,100 (5,000–31,100)	0.92
Ht (%)	28.5 (24.2–33.9)	28.8 (24.5–34.1)	25.4 (21.5–30.7)	0.06
Plt (*10000/L)	12.6 (6.8–19.2)	12.8 (7.4–19.2)	7.0 (3.9–19.9)	0.19
CRP (mg/dL)	18.0 (12.3–25.9)	18.1 (12.4–25.7)	16.2 (11.6–28.5)	0.64
T.Bil (mg/dL)	1.0 (0.7–1.7)	1.0 (0.7–1.8)	0.8 (0.5–1.5)	0.19
AST (U/L)	43 (24–103)	42 (23–103)	60 (28–143)	0.23
Alb (g/dL)	2.4 (1.9–2.7)	2.4 (2.0–2.8)	1.9 (1.7–2.6)	0.04
BUN (mg/dL)	33.5 (21.4–52.6)	33.0 (20.6–50.6)	48.0 (24.0–55.0)	0.10
Cr (mg/dL)	1.5 (1.0–2.4)	1.4 (1.0–2.2)	2.1 (1.0–3.0)	0.14
PT-INR	1.3 (1.2–1.6)	1.3 (1.1–1.5)	1.5 (1.3–2.0)	0.002
APTT (sec)	40.6 (34.0–50.9)	40.3 (33.9–49.6)	43.8 (36.7–60.7)	0.16
Lac (mmol/L)	2.6 (1.8–5.2)	2.6 (1.8–4.4)	5.6 (2.6–8.8)	< 0.001

Abbreviations: IQR, interquartile range; Alb, albumin; APTT, activated partial thromboplastin time; AST, aspartate aminotransferase; BUN, blood urea nitrogen; Cr, creatinine; CRP, C-reactive protein; HRG, histidine-rich glycoprotein; Ht, hematocrit; Lac, lactate; PCT, procalcitonin; Plt, platelet; P-SEP, presepsin; PT-INR, international normalized ratio of prothrombin time; T.Bil, total bilirubin; WBC, white blood cell.
