# Supplementary material for: Consistently low levels of histidine-rich glycoprotein as a new prognostic biomarker for sepsis: A multicenter prospective observational study
Source: PLoS One. 2023 Mar 29;18(3):e0283426. doi: 10.1371/journal.pone.0283426 (PMC10057827; doi:10.1371/journal.pone.0283426)
Supplement: S2 Table — (PDF) [file pone.0283426.s002.pdf]

S2 Table. Plasma HRG levels on days 1, 3, 5, 7, and LOCF in survivors and non-survivors [mean (95% CI)].

Time	Survivors (n = 177)	Non-survivors (n = 23)	P value
Day 1 (µg/mL)	20.7 (19.5–21.9)	15.7 (13.4–18.1)	0.006
Day 3 (µg/mL)	19.6 (18.5–20.7)	14.3 (10.9–17.7)	0.001
Day 5 (µg/mL)	20.9 (19.5–22.3)	15.9 (9.9–21.8)	0.004
Day 7 (µg/mL)	21.4 (19.8–22.9)	15.7 (11.1–20.2)	0.02
LOCF (µg/mL)	22.2 (21.0-23.4)	14.7 (12.1–17.3)	< 0.001

Abbreviations: CI, confidence interval; HRG, histidine-rich glycoprotein; LOCF, last observation till day seven carried forward.
