# Supplementary material for: Consistently low levels of histidine-rich glycoprotein as a new prognostic biomarker for sepsis: A multicenter prospective observational study
Source: PLoS One. 2023 Mar 29;18(3):e0283426. doi: 10.1371/journal.pone.0283426 (PMC10057827; doi:10.1371/journal.pone.0283426)
Supplement: S1 Fig — The Cox proportional hazards model with time-dependent covariates was used to evaluate associations between HRG levels and 28-day mortality. Higher HRG levels were significantly associated with a lower risk of mortality in all subgroups, except in the group with bilirubin levels ≥ 2 mg/dL. (PDF) [file pone.0283426.s003.pdf]

		N	HR (95% CI)
APACHE II score*	< 25	94	0.84 (0.72–0.98)
	≥ 25	106	0.87 (0.79–0.96)
Septic shock	-	68	0.64 (0.46–0.89)
	+	132	0.89 (0.82–0.97)
Creatinine*	< 2.0	135	0.89 (0.80–0.99)
	≥ 2.0	65	0.80 (0.70–0.93)
Bilirubin*	< 2.0	156	0.86 (0.79–0.94)
	≥ 2.0	44	0.70 (0.49–1.01)
Lung infection	-	149	0.80 (0.70–0.91)
	+	51	0.85 (0.76–0.96)

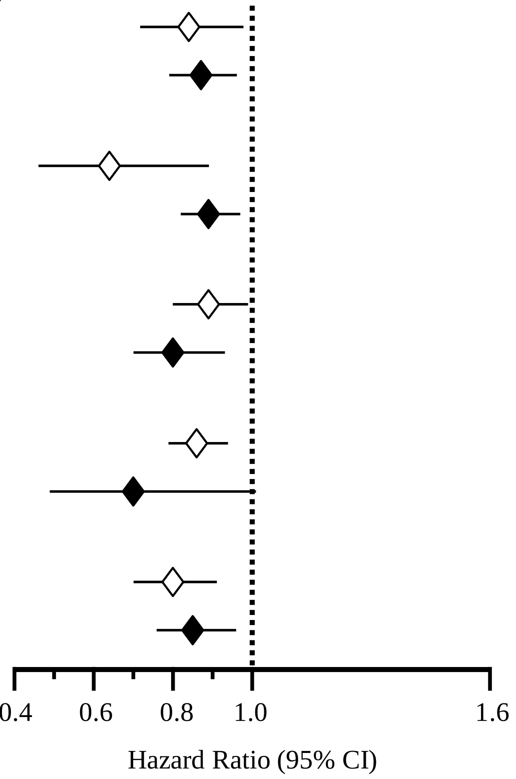

* On day 1

S1 Fig. Association between plasma HRG levels and mortality in subgroups.

Abbreviations: APACHE, Acute Physiology and Chronic Health Evaluation; CI, confidence interval; HR, hazard ratio; HRG, histidine-rich glycoprotein.
