# Supplementary material for: Consistently low levels of histidine-rich glycoprotein as a new prognostic biomarker for sepsis: A multicenter prospective observational study
Source: PLoS One. 2023 Mar 29;18(3):e0283426. doi: 10.1371/journal.pone.0283426 (PMC10057827; doi:10.1371/journal.pone.0283426)
Supplement: S2 Fig — Patients with sepsis were divided into high- and low-HRG groups according to the cutoff level of 14.04 μg/mL. The sensitivity and specificity of first-day HRG levels associated with mortality were 0.48 and 0.83, respectively, at this cutoff level. (PDF) [file pone.0283426.s004.pdf]

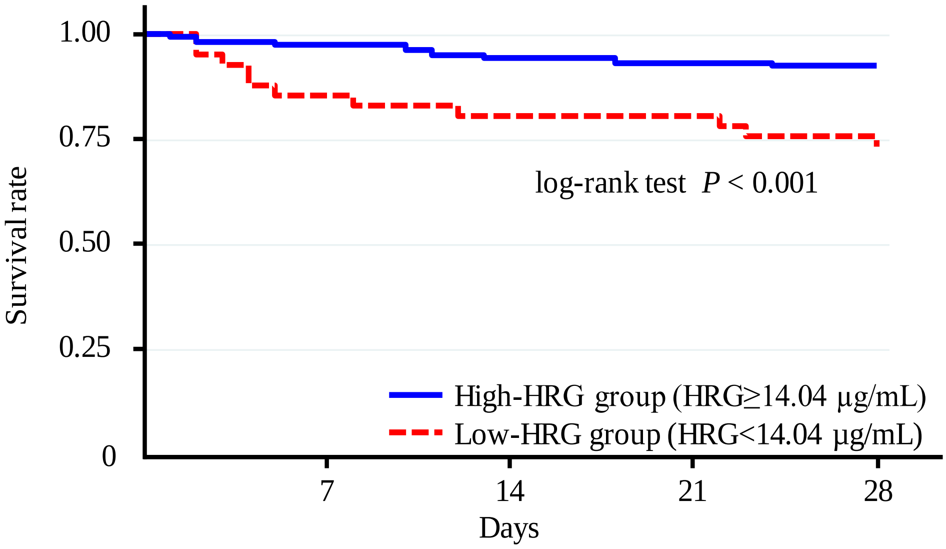

Number at risk					
High-HRG group	159	155	150	148	147
Low-HRG group	41	35	33	33	30

S2 Fig. Kaplan–Meier survival curves.

Abbreviation: HRG, histidine-rich glycoprotein.
