# Supplementary material for: Consistently low levels of histidine-rich glycoprotein as a new prognostic biomarker for sepsis: A multicenter prospective observational study
Source: PLoS One. 2023 Mar 29;18(3):e0283426. doi: 10.1371/journal.pone.0283426 (PMC10057827; doi:10.1371/journal.pone.0283426)
Supplement: S3 Fig — (PDF) [file pone.0283426.s005.pdf]

S3 Fig. Schematic diagram of enzyme-linked immunosorbent assay.

1. Solid phase (100µl, 37°C, 1hr)
Rat monoclonal antibody 3µg against human HRG (in-house, #75-14) diluted with 0.05M Na_2CO_3.

↓ **Washing** (200µl, 3 times) 0.05%Tween20-PBS

2. Blocking (150µl, 37°C, 1hr)
3%BSA-PBS.

↓ **Washing** (200µl, 3 times) 0.05%Tween20-PBS

3. Sample or Standard HRG (100µl, 20–25°C, 2hr with shaking at 500rpm)
Samples diluted 200-and 400-fold with 1%BSA-PBS.
Standards diluted purified HRG with 1%BSA-PBS (0, 0.03125, 0.0625, 0.125, 0.25, 0.5 µg/ml).

↓ **Washing** (200µl, 3 times) 0.05%Tween20-PBS

4. Detection (100µl, 20–25°C, 1hr with shaking at 500rpm)
HisProbe™-HRP diluted 1000-fold with 0.05%Tween20-PBS.

↓ **Washing** (200µl, 6 times) 0.05%Tween20-PBS

5. Labeling (200µl, 20–25°C, 10min in the dark)
SIGMAFAST™ OPD tablet.

↓ **Stop solution** 50µl, 3M H_2SO_4

6. Measuring
Measuring with plate reader using a 492 nm filter.

Abbreviations: BSA, bovine serum albumin; HRG, histidine-rich glycoprotein; HRP, horseradish peroxidase; OPD, o-phenylenediamine dihydrochloride; PBS, phosphate-buffered saline.
