# Supplementary material for: Consistently low levels of histidine-rich glycoprotein as a new prognostic biomarker for sepsis: A multicenter prospective observational study
Source: PLoS One. 2023 Mar 29;18(3):e0283426. doi: 10.1371/journal.pone.0283426 (PMC10057827; doi:10.1371/journal.pone.0283426)
Supplement: S1 Data — (DOCX) [file pone.0283426.s006.docx]

| Patient | Outcome | Study time | HRG | | | | P-SEP | | | | PCT | | | | CRP | | | | SOFA | | | |
| --- | --- | --- | --- | --- | --- | --- | --- | --- | --- | --- | --- | --- | --- | --- | --- | --- | --- | --- | --- | --- | --- | --- |
|  |  |  | Day1 | Day3 | Day5 | Day7 | Day1 | Day3 | Day5 | Day7 | Day1 | Day3 | Day5 | Day7 | Day1 | Day3 | Day5 | Day7 | Day1 | Day3 | Day5 | Day7 |
| 1 | survival | 28 | 37.14 | 25.73 |  |  | 942 | 233 |  |  | 5.97 | 1.69 |  |  | 10.59 | 15.79 |  |  | 5 | 2 |  |  |
| 2 | survival | 28 | 13.65 | 15.50 | 23.04 | 26.21 | 649 | 1050 | 525 | 353 | 5.03 | 4.86 | 0.85 | 0.32 | 8.75 | 7.89 | 4.24 | 2.05 | 12 | 11 | 9 | 6 |
| 3 | death | 23 | 14.03 | 14.49 | 11.90 | 12.62 | 2370 | 2740 | 2180 | 2280 | 8.45 | 3.26 | 2.03 | 1.21 | 13.07 | 18.97 | 8.49 | 7.56 | 12 | 16 | 14 | 16 |
| 4 | survival | 28 | 42.51 | 25.30 |  |  | 1790 | 1140 |  |  | 60.2 | 36.7 |  |  | 31.27 | 10.33 |  |  | 10 | 3 |  |  |
| 5 | death | 12 | 12.81 | 14.46 |  |  | 5470 | 5640 |  |  | 1.73 | 0.97 |  |  | 17.38 | 6.07 |  |  | 7 | 6 |  |  |
| 6 | survival | 28 | 9.14 |  |  |  | 2810 |  |  |  | 7.64 |  |  |  | 9.71 |  |  |  | 11 |  |  |  |
| 7 | survival | 28 | 27.27 | 16.23 | 12.29 | 14.80 | 7860 | 6890 | 5660 | 6600 | 100 | 60.7 | 23.1 | 18.2 | 12.46 | 25.1 | 8.5 | 5.17 | 16 | 22 | 19 | 14 |
| 8 | survival | 28 | 14.94 | 20.63 | 17.65 |  | 406 | 175 | 179 |  | 1.29 | 0.34 | 0.12 |  | 18.38 | 5.34 | 1.68 |  | 10 | 7 | 4 |  |
| 9 | survival | 28 | 22.81 |  |  |  | 423 |  |  |  | 6.17 |  |  |  | 24.94 |  |  |  | 2 |  |  |  |
| 10 | survival | 28 | 17.13 | 19.30 | 23.99 | 22.37 | 710 | 1450 | 415 | 311 | 2.22 | 1.37 | 0.69 | 0.6 | 24.31 | 9.22 | 4.83 | 2.34 | 7 | 8 | 6 | 3 |
| 11 | survival | 28 | 22.22 | 13.98 | 13.94 | 20.55 | 2530 | 2880 | 561 | 570 | 100 | 29.1 | 7.68 | 2.3 | 15.41 | 40.6 | 11.31 | 5.28 | 12 | 11 | 10 | 7 |
| 12 | survival | 28 | 11.64 | 10.49 | 14.40 | 18.36 | 4080 | 2600 | 3560 | 4830 | 2.01 | 1.15 | 0.86 | 85.6 | 0.77 | 4.55 | 5.24 | 3.93 | 12 | 7 | 7 | 12 |
| 13 | survival | 28 | 18.86 | 25.42 | 21.49 | 32.12 | 1530 | 730 | 368 | 256 | 67.7 | 11 | 2.85 | 0.8 | 13.96 | 7.51 | 2.11 | 0.93 | 6 | 6 | 2 | 1 |
| 14 | survival | 28 | 18.47 | 19.94 | 25.70 | 20.29 | 428 | 204 | 253 | 266 | 0.21 | 0.07 | 0.05 | 0.07 | 12.35 | 4.87 | 3.09 | 2.37 | 6 | 4 | 3 | 4 |
| 15 | survival | 28 | 25.69 | 30.09 |  |  | 1060 | 557 |  |  | 21.9 | 6.03 |  |  | 27.07 | 15.63 |  |  | 11 | 4 |  |  |
| 16 | survival | 28 | 15.07 | 10.06 | 9.25 | 13.26 | 1950 | 1320 | 1370 | 2280 | 58.3 | 27 | 8.57 | 2.08 | 14.54 | 22.35 | 6.47 | 5.53 | 12 | 10 | 11 | 11 |
| 17 | survival | 28 | 29.77 | 29.36 | 22.02 | 20.16 | 16000 | 10300 | 8270 | 7340 | 1.84 | 2.04 | 0.7 | 0.53 | 8.12 | 9.39 | 5.77 | 3.96 | 12 | 10 | 10 | 10 |
| 18 | survival | 28 | 11.78 | 12.36 | 11.58 | 10.77 | 922 | 1230 | 742 | 876 | 10.8 | 6.6 | 1.06 | 0.67 | 23.7 | 9.95 | 3.92 | 5.31 | 15 | 12 | 8 | 7 |
| 19 | survival | 28 | 14.32 | 13.82 | 18.26 | 28.83 | 2390 | 717 | 1470 | 848 | 72.5 | 78.3 | 38.7 | 10.7 | 26.7 | 9.38 | 10.39 | 5.8 | 9 | 12 | 6 | 3 |
| 20 | survival | 28 | 21.60 | 20.60 | 28.06 | 24.25 | 715 | 296 | 215 | 213 | 34.6 | 5.44 | 3.01 | 1.03 | 16.58 | 8.58 | 3.49 | 1.36 | 10 | 6 | 2 | 3 |
| 21 | survival | 28 | 15.00 | 17.36 | 14.36 | 22.61 | 4160 | 2390 | 1380 | 539 | 3.86 | 1.35 | 0.7 | 0.3 | 19.9 | 11.21 | 2.62 | 0.97 | 3 | 4 | 4 | 3 |
| 22 | survival | 28 | 24.61 | 21.59 | 32.44 | 27.54 | 223 | 407 | 628 | 860 | 0.4 | 0.24 | 0.11 | 0.09 | 11.41 | 5.21 | 8.14 | 8.56 | 8 | 7 | 5 | 5 |
| 23 | survival | 28 | 28.41 | 24.09 | 13.33 | 16.85 | 934 | 1120 | 2220 | 2420 | 4.14 | 2.7 | 4.43 | 2.1 | 28.23 | 10.99 | 21.55 | 13.22 | 12 | 7 | 14 | 14 |
| 24 | survival | 28 | 18.28 | 15.95 | 13.31 | 12.78 | 1320 | 4150 | 9410 | 7390 | 0.6 | 0.82 | 0.88 | 0.61 | 11.77 | 8.98 | 10.68 | 6.79 | 6 | 6 | 8 | 9 |
| 25 | survival | 28 | 20.53 | 17.75 |  |  | 542 | 327 |  |  | 54.4 | 13 |  |  | 25.56 | 10.49 |  |  | 9 | 5 |  |  |
| 26 | death | 13 | 23.51 | 14.31 | 13.40 | 14.40 | 628 | 720 | 739 | 713 | 0.29 | 0.1 | 0.09 | 0.08 | 0.39 | 0.71 | 0.25 | 0.2 | 10 | 10 | 7 | 7 |
| 27 | survival | 28 | 21.41 | 21.60 | 19.54 |  | 2980 | 2030 | 1290 |  | 0.27 | 0.24 | 0.21 |  | 10.97 | 7.47 | 7.2 |  | 3 | 3 | 3 |  |
| 28 | survival | 28 | 18.92 | 19.90 | 20.53 | 16.51 | 2360 | 2470 | 1990 | 1740 | 1.88 | 1.58 | 0.4 | 0.28 | 16.09 | 5.85 | 2.87 | 1.28 | 9 | 13 | 11 | 11 |
| 29 | survival | 28 | 30.20 |  |  |  | 1260 |  |  |  | 50 |  |  |  | 30.46 |  |  |  | 6 |  |  |  |
| 30 | survival | 28 | 13.27 | 12.84 | 11.13 | 11.85 | 1230 | 841 | 1230 | 1400 | 10.7 | 4 | 7.31 | 12.4 | 13.45 | 9.37 | 4.84 | 7.41 | 13 | 13 | 12 | 9 |
| 31 | survival | 28 | 29.43 | 25.35 |  |  | 2120 | 1820 |  |  | 100 | 27.6 |  |  | 22.02 | 8.89 | 2.36 |  | 9 | 10 | 6 |  |
| 32 | survival | 28 | 18.54 | 18.32 | 19.68 | 19.77 | 1230 | 672 | 842 | 1670 | 1.04 | 0.24 | 0.19 | 0.22 | 8.1 | 5.59 | 3.83 | 4.13 | 10 | 1 | 0 | 0 |
| 33 | survival | 28 | 9.78 |  |  |  | 2500 |  |  |  | 14.9 |  |  |  | 29.48 |  |  |  | 9 |  |  |  |
| 34 | survival | 28 | 19.06 | 14.48 |  |  | 1350 | 1680 |  |  | 48.5 | 17.3 |  |  | 22.32 | 29.47 |  |  | 8 | 2 |  |  |
| 35 | survival | 28 | 13.60 | 14.17 | 15.31 | 10.75 | 884 | 722 | 862 | 1410 | 7.74 | 2.59 | 0.66 | 19.3 | 37 | 11.88 | 5.33 | 19.06 | 12 | 5 | 5 | 7 |
| 36 | survival | 28 | 14.76 | 12.42 | 12.88 | 11.45 | 549 | 741 | 725 | 692 | 7.68 | 5.49 | 3.79 | 1.71 | 17.77 | 17.18 | 13.1 | 13.91 | 10 | 5 | 5 | 3 |
| 37 | survival | 28 | 10.33 | 6.79 | 7.77 | 13.32 | 1560 | 1760 | 2200 | 2260 | 6.61 | 11.7 | 4.47 | 1.53 | 13.22 | 17.32 | 4.94 | 8.77 | 15 | 18 | 20 | 15 |
| 38 | survival | 28 | 22.92 | 23.61 | 15.20 |  | 259 | 335 | 273 |  | 19.7 | 5.44 | 1.61 |  | 16.78 | 10.61 | 2.27 |  | 10 | 7 | 5 |  |
| 39 | survival | 28 | 18.42 | 20.32 |  |  | 272 | 500 |  |  | 3.83 | 1.13 |  |  | 29.13 | 23.48 |  |  | 12 | 3 |  |  |
| 40 | survival | 28 | 15.82 | 19.88 | 18.71 | 16.83 | 2400 | 1170 | 652 | 624 | 64.9 | 43.2 | 12 | 2.97 | 27.13 | 9.7 | 3.5 | 3.46 | 13 | 5 | 5 | 5 |
| 41 | survival | 28 | 32.11 | 23.51 |  |  | 298 | 231 |  |  | 0.5 | 0.15 |  |  | 1.3 | 0.6 |  |  | 6 | 0 |  |  |
| 42 | survival | 28 | 18.40 | 13.01 |  |  | 1140 | 717 |  |  | 20.1 | 10.3 |  |  | 34.15 | 23.3 |  |  | 7 | 3 |  |  |
| 43 | survival | 28 | 20.84 | 13.78 |  |  | 1680 | 772 |  |  | 100 | 59.8 |  |  | 9.57 | 22.65 |  |  | 11 | 5 |  |  |
| 44 | survival | 28 | 24.44 | 25.65 | 22.81 | 31.24 | 776 | 771 | 531 | 420 | 0.06 | 0.09 | 0.06 | 0.15 | 3.44 | 4.26 | 0.71 | 0.08 | 7 | 4 | 3 | 3 |
| 45 | survival | 28 | 16.08 | 13.48 | 23.66 | 19.60 | 571 | 526 | 231 | 245 | 5.58 | 1.42 | 0.25 | 0.08 | 2.46 | 8.43 | 1.56 | 0.67 | 10 | 7 | 7 | 4 |
| 46 | survival | 28 | 18.51 | 18.36 |  |  | 305 | 258 |  |  | 23.9 | 9.24 |  |  | 12.33 | 9.04 | 5.82 | 5.47 | 6 | 3 | 1 | 1 |
| 47 | survival | 28 | 24.58 | 25.02 | 33.68 | 34.26 | 5110 | 1350 | 490 | 459 | 16.5 | 10.3 | 2.62 | 1.04 | 19.29 | 23.27 | 5.76 | 2.38 | 12 | 9 | 5 | 2 |
| 48 | survival | 28 | 22.37 | 13.58 | 16.78 | 16.28 | 1300 | 1150 | 843 | 514 | 50.9 | 39 | 19.3 | 5.64 | 15.23 | 20.87 | 2.72 | 1.1 | 12 | 8 | 0 | 1 |
| 49 | survival | 28 | 18.27 | 20.27 |  |  | 531 | 238 |  |  | 51.7 | 22.2 |  |  | 23.59 | 5.24 |  |  | 10 | 6 |  |  |
| 50 | survival | 28 | 16.60 | 10.95 | 12.74 |  | 585 | 780 | 331 |  | 78.9 | 29.1 | 8.69 |  | 25.96 | 35.4 | 26.95 |  | 5 | 4 | 3 |  |
| 51 | survival | 28 | 15.13 | 15.77 | 16.21 | 16.44 | 1950 | 1050 | 576 | 956 | 67.8 | 39.9 | 15.3 | 4.07 | 10.67 | 24.11 | 13.96 | 7.84 | 12 | 8 | 7 | 7 |
| 52 | survival | 28 | 15.86 | 24.43 |  |  | 412 | 374 |  |  | 11.9 | 4.39 |  |  | 25.6 | 18.55 |  |  | 5 | 1 |  |  |
| 53 | survival | 28 | 20.46 | 18.88 | 33.63 |  | 352 | 376 | 194 |  | 0.56 | 0.38 | 0.17 |  | 18.09 | 15.13 | 6.84 |  | 8 | 1 | 2 |  |
| 54 | survival | 28 | 15.50 | 14.40 | 25.17 | 17.93 | 965 | 457 | 322 | 328 | 14.5 | 6.81 | 2.78 | 0.96 | 6.17 | 2.86 | 1.71 | 0.74 | 4 | 3 | 3 | 2 |
| 55 | death | 10 | 14.99 | 14.18 | 18.40 | 22.57 | 653 | 415 | 363 | 415 | 2.17 | 4.09 | 0.93 | 0.22 | 8.64 | 5.78 | 3.54 | 1.87 | 12 | 5 | 5 | 5 |
| 56 | survival | 28 | 12.80 | 12.87 |  |  | 12200 | 13300 |  |  | 4.73 | 3.96 |  |  | 8.73 | 6.17 | 4.14 |  | 9 | 7 | 7 |  |
| 57 | survival | 28 | 16.20 |  |  |  | 324 |  |  |  | 14.2 |  |  |  | 24 |  |  |  | 2 |  |  |  |
| 58 | survival | 28 | 11.37 | 6.95 | 11.32 | 21.32 | 1350 | 1210 | 2500 | 717 | 28.1 | 26.5 | 13.4 | 3.8 | 25.24 | 21.79 | 8 | 3.75 | 15 | 19 | 10 | 7 |
| 59 | survival | 28 | 7.93 | 8.88 | 9.61 |  | 14000 | 12200 | 8700 |  | 0.6 | 0.57 | 0.28 |  | 14.82 | 10.7 | 6.12 |  | 13 | 11 | 10 |  |
| 60 | survival | 28 | 26.83 | 25.16 | 27.44 | 14.34 | 1650 | 554 | 422 | 116 | 100 | 37.4 | 10.1 | 1.48 | 34.7 | 11.54 | 3.28 | 1.43 | 11 | 5 | 3 | 1 |
| 61 | death | 18 | 19.12 | 11.89 | 18.09 | 21.37 | 1380 | 742 | 699 | 552 | 5.58 | 1.55 | 0.24 | 0.12 | 28.49 | 15.36 | 10.62 | 7.61 | 12 | 7 | 5 | 5 |
| 62 | survival | 28 | 22.45 | 14.95 | 14.17 | 18.94 | 372 | 472 | 852 | 690 | 17.4 | 50.2 | 16.8 | 5.73 | 12.51 | 26.69 | 24.9 | 20.23 | 10 | 9 | 5 | 3 |
| 63 | survival | 28 | 14.43 | 22.62 | 26.52 | 21.36 | 6880 | 9610 | 3840 | 2020 | 100 | 59.9 | 8.72 | 2.47 | 39.16 | 22.71 | 9.79 | 9.36 | 15 | 9 | 7 | 5 |
| 64 | survival | 28 | 16.28 | 17.85 |  |  | 2330 | 1420 |  |  | 100 | 77 |  |  | 14.49 | 4.11 |  |  | 10 | 6 |  |  |
| 65 | survival | 28 | 18.17 | 16.54 | 20.24 | 19.86 | 1340 | 1730 | 871 | 1060 | 6.29 | 3.34 | 0.63 | 0.24 | 40.04 | 27.95 | 9.63 | 8.71 | 6 | 2 | 1 | 1 |
| 66 | survival | 28 | 17.54 | 15.10 |  |  | 5440 | 4990 |  |  | 8.82 | 16.7 |  |  | 33.36 | 25.53 |  |  | 12 | 9 |  |  |
| 67 | survival | 28 | 10.20 | 12.01 | 27.25 |  | 1190 | 566 | 211 |  | 100 | 78.7 | 0.93 |  | 16.55 | 8.57 | 6.25 | 2.17 | 13 | 7 | 4 | 1 |
| 68 | survival | 28 | 14.97 | 10.75 | 20.38 |  | 1450 | 1370 | 1180 |  | 41.6 | 19.1 | 4.5 |  | 15.88 | 35.34 | 13.6 |  | 12 | 6 | 6 |  |
| 69 | survival | 28 | 21.21 | 18.56 | 19.76 | 18.67 | 409 | 239 | 208 | 190 | 0.25 | 0.12 | 0.06 | 0.05 | 20.78 | 5.51 | 3.7 | 1.42 | 15 | 5 | 3 | 2 |
| 70 | survival | 28 | 13.84 |  |  |  | 1470 |  |  |  | 4.67 |  |  |  | 11.63 | 14.76 |  |  | 8 | 5 |  |  |
| 71 | survival | 28 | 17.16 | 17.02 | 16.55 | 20.41 | 521 | 640 | 380 | 363 | 8.08 | 3.35 | 0.9 | 0.4 | 21.3 | 13.49 | 10.57 | 9.23 | 10 | 5 | 3 | 2 |
| 72 | death | 18 | 16.23 | 10.43 | 14.32 | 16.20 | 767 | 1350 | 2210 | 3440 | 13.2 | 7.72 | 4.41 | 3 | 16.24 | 9.74 | 6.28 | 2.99 | 13 | 12 | 14 | 9 |
| 73 | survival | 28 | 20.56 | 23.39 |  |  | 247 | 364 |  |  | 1.97 | 0.89 |  |  | 1.93 | 6.79 |  |  | 9 | 5 |  |  |
| 74 | survival | 28 | 48.33 | 49.95 |  |  | 256 | 224 |  |  | 2.72 | 3.34 |  |  | 5.87 | 4.68 |  |  | 5 | 5 |  |  |
| 75 | survival | 28 | 46.37 | 41.95 |  |  | 592 | 286 |  |  | 4.4 | 1.99 |  |  | 3.98 | 14.14 |  |  | 7 | 3 |  |  |
| 76 | survival | 28 | 12.09 | 13.88 | 24.18 | 21.39 | 17500 | 12700 | 12600 | 6030 | 16.9 | 8.16 | 4.62 | 1.78 | 20.88 | 13.51 | 8.04 | 3.66 | 19 | 19 | 15 | 10 |
| 77 | survival | 28 | 19.65 | 18.06 |  |  | 534 | 443 |  |  | 20.9 | 13.8 |  |  | 20.89 | 19.3 |  |  | 3 | 1 |  |  |
| 78 | survival | 28 | 23.78 | 21.89 | 23.45 | 26.51 | 471 | 321 | 232 | 224 | 29.9 | 7.34 | 2.23 | 0.9 | 21.01 | 17.59 | 7.8 | 7.7 | 9 | 4 | 2 | 0 |
| 79 | death | 10 | 19.47 | 11.45 |  |  | 2590 | 1520 |  |  | 82.9 | 79.4 |  |  | 18.98 | 31.58 |  |  | 8 | 5 |  |  |
| 80 | death | 28 | 8.82 | 10.70 | 12.62 |  | 1350 | 877 | 669 |  | 39.5 | 9.68 | 4.24 |  | 27.88 | 17.56 | 13.04 |  | 8 | 6 | 5 |  |
| 81 | survival | 28 | 39.96 | 42.53 | 40.95 | 38.67 | 1380 | 591 | 415 | 200 | 39.6 | 18.1 | 3.98 | 1.01 | 30.46 | 18.28 | 10.43 | 2.87 | 5 | 1 | 1 | 1 |
| 82 | survival | 28 | 21.72 | 19.44 |  |  | 3150 | 912 |  |  | 1.46 | 0.57 |  |  | 1.37 | 0.96 |  |  | 13 | 6 |  |  |
| 83 | survival | 28 | 25.45 | 22.42 |  |  | 371 | 167 |  |  | 48.3 | 13.2 |  |  | 24.97 | 14.1 |  |  | 5 | 4 |  |  |
| 84 | survival | 28 | 17.93 | 15.07 | 15.12 | 17.80 | 729 | 625 | 717 | 737 | 0.68 | 0.43 | 0.27 | 0.12 | 12.56 | 12.4 | 8.21 | 4.17 | 9 | 8 | 7 | 3 |
| 85 | survival | 28 | 23.47 | 27.27 |  |  | 1460 | 945 |  |  | 12.6 | 5.12 |  |  | 13.87 | 7.38 |  |  | 11 | 3 |  |  |
| 86 | survival | 28 | 17.33 | 29.54 |  |  | 274 | 185 |  |  | 0.86 | 0.32 |  |  | 13 | 10.36 |  |  | 9 | 6 |  |  |
| 87 | survival | 28 | 26.54 | 22.27 | 26.77 | 28.52 | 981 | 1140 | 732 | 491 | 11 | 10.4 | 3.75 | 1.02 | 20 | 23.3 | 9.75 | 6.11 | 12 | 10 | 8 | 6 |
| 88 | survival | 28 | 20.76 | 24.49 |  |  | 707 | 552 |  |  | 5.69 | 2.66 |  |  | 14.4 | 19.8 |  |  | 7 | 6 |  |  |
| 89 | survival | 28 | 12.09 | 15.35 | 24.58 |  | 3460 | 3640 | 1780 |  | 4.27 | 4.4 | 1.12 |  | 21.3 | 19.74 | 7.8 |  | 12 | 12 | 8 |  |
| 90 | survival | 28 | 23.73 | 22.69 | 25.61 | 17.01 | 413 | 249 | 233 | 268 | 0.62 | 0.57 | 0.15 | 0.11 | 15 | 9.21 | 2.83 | 3 | 10 | 9 | 8 | 8 |
| 91 | survival | 28 | 29.92 | 35.78 |  |  | 306 | 219 |  |  | 0.53 | 0.17 |  |  | 4.8 | 6 |  |  | 10 | 3 |  |  |
| 92 | death | 11 | 24.41 | 25.04 | 13.54 | 12.11 | 1570 | 1930 | 3300 | 3830 | 19.2 | 20 | 13.7 | 8.29 | 8.2 | 23.5 | 27 | 21 | 15 | 15 | 19 | 19 |
| 93 | survival | 28 | 24.64 | 17.48 | 21.49 | 19.85 | 810 | 850 | 620 | 349 | 25.2 | 19.9 | 5.94 | 1.69 | 26.7 | 24 | 15 | 8 | 8 | 7 | 7 | 6 |
| 94 | survival | 28 | 24.08 | 23.82 | 16.48 | 20.44 | 407 | 612 | 646 | 520 | 2.36 | 1.93 | 0.89 | 0.38 | 27.4 | 29.7 | 26 | 24.2 | 9 | 9 | 9 | 9 |
| 95 | survival | 28 | 24.57 | 23.02 |  |  | 409 | 299 |  |  | 6.5 | 3.67 |  |  | 21.9 | 18.7 |  |  | 9 | 7 |  |  |
| 96 | survival | 28 | 19.09 | 18.01 | 25.29 |  | 1210 | 1070 | 751 |  | 4.01 | 2.16 | 0.72 |  | 19.4 | 14 | 4.61 |  | 12 | 8 | 3 |  |
| 97 | survival | 28 | 34.72 |  |  |  | 164 |  |  |  | 10.1 |  |  |  | 12.6 |  |  |  | 3 |  |  |  |
| 98 | survival | 28 | 20.01 | 19.05 |  |  | 636 | 462 |  |  | 100 | 79.7 |  |  | 22.7 | 10.73 |  |  | 10 | 5 |  |  |
| 99 | survival | 28 | 24.46 | 27.84 |  |  | 803 | 193 |  |  | 18.8 | 4.72 |  |  | 10.7 | 16 |  |  | 8 | 7 |  |  |
| 100 | survival | 28 | 22.83 | 17.50 | 16.83 | 15.01 | 422 | 398 | 234 | 211 | 0.84 | 2.17 | 0.62 | 0.19 | 18.25 | 22.67 | 5.66 | 2.27 | 6 | 7 | 5 | 6 |
| 101 | survival | 28 | 19.97 | 20.33 | 24.17 | 22.03 | 1650 | 4060 | 4450 | 5960 | 100 | 100 | 100 | 23.3 | 25.8 | 26 | 9.8 | 4.31 | 19 | 20 | 18 | 14 |
| 102 | survival | 28 | 13.32 | 13.15 | 15.26 | 17.07 | 2260 | 998 | 511 | 381 | 15.1 | 11.6 | 2.99 | 1.19 | 29 | 32 | 8.43 | 5.51 | 10 | 8 | 6 | 1 |
| 103 | survival | 28 | 43.48 | 42.96 |  |  | 1080 | 578 |  |  | 0.32 | 0.24 |  |  | 18 | 8.63 |  |  | 7 | 4 |  |  |
| 104 | survival | 28 | 21.46 | 27.80 |  |  | 842 | 297 |  |  | 22.6 | 9.21 |  |  | 4.45 | 5.25 |  |  | 5 | 3 |  |  |
| 105 | survival | 28 | 19.03 | 23.63 |  |  | 272 | 194 |  |  | 2.79 | 1.21 |  |  | 18 | 14.35 |  |  | 3 | 2 |  |  |
| 106 | survival | 28 | 14.04 | 14.45 |  |  | 2720 | 2470 |  |  | 21.9 | 9.86 |  |  | 26.9 | 16.59 |  |  | 11 | 10 |  |  |
| 107 | survival | 28 | 12.22 | 10.86 | 14.24 | 21.49 | 760 | 675 | 324 | 308 | 32.6 | 13.7 | 3.08 | 0.7 | 10.74 | 11.85 | 2.66 | 0.84 | 12 | 9 | 8 | 4 |
| 108 | survival | 28 | 13.71 |  |  |  | 757 |  |  |  | 0.36 |  |  |  | 12 |  |  |  | 4 |  |  |  |
| 109 | survival | 28 | 11.91 | 11.99 |  |  | 1070 | 724 |  |  | 24.9 | 8.62 |  |  | 13.27 | 23.47 |  |  | 10 | 4 |  |  |
| 110 | survival | 28 | 15.84 | 15.41 | 21.88 |  | 500 | 454 | 319 |  | 12.3 | 6.93 | 2.33 |  | 30.42 | 24.7 | 11 |  | 10 | 8 | 2 |  |
| 111 | survival | 28 | 17.01 | 17.80 | 24.07 |  | 1360 | 770 | 443 |  | 52.7 | 29.2 | 14.1 |  | 26.7 | 24.53 | 7.43 |  | 14 | 11 | 10 |  |
| 112 | survival | 28 | 37.87 |  |  |  | 190 |  |  |  | 0.09 |  |  |  | 5.61 |  |  |  | 6 |  |  |  |
| 113 | survival | 28 | 28.82 | 33.32 | 27.12 | 28.44 | 1020 | 476 | 409 | 247 | 0.61 | 0.31 | 0.16 | 0.08 | 24.03 | 23.32 | 14.88 | 3.72 | 11 | 7 | 6 | 5 |
| 114 | survival | 28 | 26.18 | 26.15 |  |  | 899 | 359 |  |  | 28.9 | 9.66 |  |  | 24.42 | 10.18 |  |  | 6 | 3 |  |  |
| 115 | survival | 28 | 25.99 | 28.15 |  |  | 99 | 177 |  |  | 0 | 0.03 |  |  | 6.21 | 4.78 |  |  | 3 | 3 |  |  |
| 116 | survival | 28 | 19.80 | 20.03 | 24.79 |  | 508 | 373 | 269 |  | 0.55 | 0.37 | 0.14 |  | 8.28 | 3.72 | 1.72 |  | 7 | 4 | 4 |  |
| 117 | death | 11 | 23.62 | 30.21 | 41.20 | 28.02 | 233 | 264 | 242 | 380 | 0.36 | 0.35 | 0.2 | 0.21 | 12.29 | 18.61 | 16.91 | 15.98 | 11 | 9 | 10 | 10 |
| 118 | survival | 28 | 45.49 |  |  |  | 266 |  |  |  | 0.58 |  |  |  | 16.47 |  |  |  | 5 |  |  |  |
| 119 | survival | 28 | 23.41 | 17.30 | 21.07 |  | 353 | 344 | 275 |  | 15.2 | 3.82 | 0.74 |  | 8.17 | 2.77 | 1.4 |  | 7 | 7 | 3 |  |
| 120 | survival | 28 | 17.95 | 13.81 | 26.57 | 40.16 | 403 | 410 | 286 | 184 | 7.48 | 2.86 | 0.89 | 0.27 | 4.6 | 23.9 | 11.4 | 4.1 | 11 | 11 | 8 | 4 |
| 121 | survival | 28 | 31.99 | 22.91 |  |  | 695 | 530 |  |  | 1.6 | 1.18 |  |  | 0.09 | 11.24 |  |  | 6 | 7 |  |  |
| 122 | survival | 28 | 17.44 | 14.74 |  |  | 541 | 419 |  |  | 38.2 | 19.4 |  |  | 29.9 | 24.8 |  |  | 11 | 9 |  |  |
| 123 | survival | 28 | 20.31 |  |  |  | 395 |  |  |  | 1.79 |  |  |  | 22.6 | 22.4 |  |  | 9 | 9 |  |  |
| 124 | death | 24 | 18.81 | 12.15 | 11.10 | 11.86 | 3010 | 6820 | 12600 | 11500 | 25.5 | 90.6 | 52.6 | 20.9 | 16 | 17.23 | 8.3 | 6.23 | 17 | 19 | 19 | 20 |
| 125 | survival | 28 | 33.44 | 19.92 |  |  | 1030 | 1010 |  |  | 67.3 | 23.2 |  |  | 21.86 | 24.4 |  |  | 8 | 6 |  |  |
| 126 | survival | 28 | 31.64 | 28.03 | 32.01 |  | 266 | 322 | 189 |  | 4.64 | 3.29 | 1.24 |  | 19.63 | 23.1 | 7.23 |  | 10 | 8 | 2 |  |
| 127 | survival | 28 | 30.68 | 13.05 | 22.11 |  | 514 | 501 | 381 |  | 28.6 | 24.5 | 7.82 |  | 14.6 | 29.6 | 21.4 |  | 9 | 8 | 3 |  |
| 128 | survival | 28 | 22.27 | 25.43 |  |  | 258 | 369 |  |  | 18.1 | 6.26 |  |  | 15.9 | 5.71 |  |  | 8 | 3 |  |  |
| 129 | survival | 28 | 28.52 |  |  |  | 991 |  |  |  | 0.91 |  |  |  | 40.4 |  |  |  | 5 |  |  |  |
| 130 | survival | 28 | 25.08 | 18.56 | 23.73 | 27.88 | 502 | 564 | 585 | 478 | 28.3 | 40.1 | 7.02 | 1.72 | 22.75 | 26.5 | 24.8 | 12.4 | 3 | 5 | 10 | 5 |
| 131 | survival | 28 | 19.19 | 17.17 | 18.13 |  | 281 | 261 | 230 |  | 75.9 | 56.4 | 14.8 |  | 20.3 | 19.8 | 8.8 |  | 10 | 5 | 3 |  |
| 132 | survival | 28 | 32.54 | 34.11 | 39.86 |  | 350 | 189 | 140 |  | 1.09 | 0.31 | 0.17 |  | 3.25 | 9 | 2.02 |  | 10 | 8 | 4 |  |
| 133 | survival | 28 | 13.82 | 17.88 | 19.62 | 18.60 | 2550 | 2330 | 1180 | 2280 | 4.22 | 2.49 | 0.66 | 0.28 | 24.25 | 22.19 | 8.75 | 5.54 | 10 | 7 | 6 | 5 |
| 134 | survival | 28 | 11.60 | 13.65 | 17.43 | 19.79 | 2520 | 1070 | 879 | 525 | 87.2 | 46.2 | 12.9 | 4.72 | 17.59 | 10.37 | 3.34 | 1.61 | 3 | 4 | 1 | 0 |
| 135 | survival | 28 | 14.08 | 12.68 | 13.92 | 16.92 | 1350 | 2420 | 2180 | 1920 | 13.4 | 7.35 | 3.3 | 1.32 | 3.18 | 33.77 | 16.05 | 14.54 | 15 | 9 | 10 | 8 |
| 136 | survival | 28 | 26.14 | 24.47 | 28.05 | 24.34 | 393 | 239 | 208 | 243 | 9.61 | 4.53 | 1.29 | 0.38 | 19.72 | 7.91 | 4.58 | 4.67 | 5 | 5 | 2 | 1 |
| 137 | survival | 28 | 14.93 | 14.68 | 19.86 | 22.87 | 1190 | 677 | 486 | 338 | 0.94 | 0.38 | 0.12 | 0.06 | 8.28 | 17.25 | 5.08 | 1.54 | 13 | 5 | 6 | 5 |
| 138 | survival | 28 | 18.41 | 22.26 | 30.40 | 34.75 | 1620 | 561 | 575 | 345 | 65 | 72 | 21.2 | 6.15 | 30.61 | 19.57 | 9.05 | 3.39 | 7 | 4 | 2 | 1 |
| 139 | survival | 28 | 17.51 | 15.79 | 18.00 | 24.35 | 3600 | 1850 | 770 | 536 | 32.5 | 22 | 4.2 | 1.33 | 31 | 10.92 | 5.52 | 1.28 | 16 | 6 | 5 | 4 |
| 140 | death | 1 | 18.91 |  |  |  | 740 |  |  |  | 12.7 |  |  |  | 5.22 |  |  |  | 16 |  |  |  |
| 141 | death | 5 | 21.75 | 24.71 |  |  | 1510 | 614 |  |  | 6.42 | 1.97 |  |  | 30.5 | 13.93 | 21.06 |  | 12 | 9 | 15 |  |
| 142 | survival | 28 | 16.22 | 16.27 | 20.46 | 19.79 | 9840 | 14800 | 11600 | 13000 | 92.5 | 100 | 61.4 | 23.4 | 5.19 | 11.96 | 6.31 | 4.98 | 11 | 19 | 15 | 7 |
| 143 | death | 2 | 11.16 |  |  |  | 1080 |  |  |  | 62.6 |  |  |  | 28.65 |  |  |  | 16 |  |  |  |
| 144 | survival | 28 | 23.56 | 27.98 | 29.36 | 38.71 | 10800 | 7370 | 2200 | 745 | 100 | 100 | 24.7 | 5.03 | 17.34 | 13.55 | 7.66 | 5.01 | 13 | 10 | 9 | 6 |
| 145 | survival | 28 | 25.15 | 29.18 | 44.41 | 36.72 | 1220 | 310 | 183 | 163 | 3.5 | 0.81 | 0.19 | 0.08 | 17.75 | 3.27 | 1.21 | 0 | 8 | 2 | 1 |  |
| 146 | survival | 28 | 18.22 | 14.30 | 14.27 |  | 1190 | 1370 | 1050 |  | 77.5 | 58.1 | 19.7 |  | 16.99 | 28.08 | 25.07 | 19.42 | 15 | 11 | 6 | 2 |
| 147 | survival | 28 | 35.00 | 24.38 |  |  | 351 | 294 |  |  | 64.3 | 36.5 |  |  | 7.11 | 5.97 |  |  | 6 | 2 |  |  |
| 148 | survival | 28 | 27.03 |  |  |  | 720 |  |  |  | 20.2 |  |  |  | 13.65 |  |  |  | 5 |  |  |  |
| 149 | survival | 28 | 18.08 | 22.70 | 32.53 | 32.34 | 558 | 517 | 282 | 255 | 1.72 | 1.69 | 0.63 | 0.22 | 12.46 | 14.84 | 7.16 | 2.26 | 4 | 6 | 5 | 1 |
| 150 | death | 4 | 13.59 | 15.18 |  |  | 1130 | 2150 |  |  | 97.4 | 100 |  |  | 16.35 | 24.89 |  |  | 13 | 18 |  |  |
| 151 | survival | 28 | 20.22 | 42.14 |  |  | 943 | 525 |  |  | 1.8 | 3.76 |  |  | 25.97 | 19.67 |  |  | 2 | 2 |  |  |
| 152 | death | 2 | 19.89 |  |  |  | 277 |  |  |  | 0.78 |  |  |  | 5.5 |  |  |  | 13 |  |  |  |
| 153 | survival | 28 | 59.88 | 40.20 | 40.62 | 23.67 | 930 | 1130 | 591 | 405 | 0.47 | 2.43 | 0.71 | 0.18 | 24.45 | 38.13 | 0.35 | 2 | 8 | 10 | 5 | 2 |
| 154 | survival | 28 | 8.56 | 17.00 |  |  | 593 | 671 |  |  | 19.1 | 5.18 |  |  | 45.54 | 35.02 |  |  | 14 | 8 |  |  |
| 155 | death | 2 | 21.05 |  |  |  | 3240 |  |  |  | 37.1 |  |  |  | 35 |  |  |  | 14 |  |  |  |
| 156 | survival | 28 | 30.64 | 27.68 |  |  | 4380 | 1250 |  |  | 30.6 | 8.89 |  |  | 10.19 | 13.83 |  |  | 8 | 7 |  |  |
| 157 | survival | 28 | 7.29 | 6.38 | 7.11 | 5.82 | 2270 | 3240 | 3060 | 3040 | 100 | 80.6 | 33.2 | 12.5 | 2.35 | 14.77 | 12.11 | 5.26 | 16 | 14 | 15 | 14 |
| 158 | survival | 28 | 22.14 | 21.33 | 20.94 | 26.34 | 288 | 243 | 219 | 143 | 8.61 | 3.35 | 1.1 | 0.39 | 30.31 | 27.53 | 5.88 | 5.75 | 15 | 9 | 2 | 2 |
| 159 | death | 22 | 6.72 | 9.92 | 10.73 | 9.77 | 1590 | 2710 | 2150 | 3490 | 58.1 | 40.2 | 18.8 | 22.7 | 37.05 | 28.14 | 29.9 | 31.66 | 13 | 14 | 15 | 16 |
| 160 | survival | 28 | 8.66 | 12.74 | 14.09 | 13.64 | 4780 | 4920 | 5670 | 6060 | 59.7 | 46.6 | 22.1 | 55.8 | 30 | 15.99 | 10.22 | 7.59 | 13 | 9 | 9 | 8 |
| 161 | survival | 28 | 16.53 | 17.19 |  |  | 8690 | 4010 |  |  | 15.1 | 6.1 |  |  | 18.08 | 17.05 |  |  | 13 | 11 |  |  |
| 162 | survival | 28 | 17.48 | 16.14 | 17.43 | 18.19 | 1350 | 1230 | 720 | 388 | 78.2 | 24.4 | 4.42 | 0.88 | 23.34 | 14.9 | 7.44 | 2.91 | 9 | 5 | 3 | 2 |
| 163 | survival | 28 | 21.40 | 18.67 | 24.17 | 19.70 | 6650 | 1970 | 1020 | 470 | 68.3 | 88.1 | 23.6 | 6.78 | 38.84 | 26.72 | 3.51 | 2.49 | 13 | 11 | 8 | 6 |
| 164 | death | 3 | 12.36 |  |  |  | 5310 |  |  |  | 63.6 |  |  |  | 12.63 |  |  |  | 21 |  |  |  |
| 165 | death | 4 | 10.96 | 4.60 |  |  | 2050 | 2230 |  |  | 3.38 | 1.19 |  |  | 20.42 | 5.09 |  |  | 17 | 18 |  |  |
| 166 | survival | 28 | 31.38 | 18.20 | 35.50 | 24.50 | 2150 | 1810 | 1650 | 1490 | 100 | 43.5 | 13.8 | 5.4 | 38.88 | 40.11 | 24.74 | 26.08 | 7 | 5 | 2 | 1 |
| 167 | survival | 28 | 17.23 | 19.56 | 17.88 | 20.53 | 1000 | 401 | 529 | 1010 | 1.4 | 0.74 | 0.23 | 0.37 | 24.3 | 11.2 | 7.61 | 12.76 | 4 | 3 | 3 | 3 |
| 168 | survival | 28 | 17.45 | 15.58 |  |  | 991 | 408 |  |  | 28.2 | 5.32 |  |  | 45.36 | 31.4 |  |  | 8 | 5 |  |  |
| 169 | survival | 28 | 23.39 | 16.33 | 17.35 | 22.89 | 992 | 484 | 424 | 415 | 5.01 | 1.45 | 0.3 | 0.13 | 15.37 | 9.38 | 7.79 | 3.92 | 12 | 9 | 8 | 7 |
| 170 | death | 8 | 10.04 | 10.27 | 9.37 | 8.04 | 4050 | 3220 | 3930 | 3100 | 40.2 | 19.5 | 12.1 | 6.96 | 13.77 | 9.14 | 7.92 | 4.54 | 12 | 10 | 10 | 17 |
| 171 | survival | 28 | 16.05 | 12.64 | 23.18 | 28.22 | 3610 | 724 | 2050 | 685 | 93.1 | 23.2 | 6.41 | 1.61 | 21.02 | 8.76 | 2.23 | 1.24 | 13 | 7 | 8 | 5 |
| 172 | survival | 28 | 22.44 | 17.07 | 18.81 | 24.05 | 652 | 464 | 353 | 321 | 14.1 | 2.87 | 0.87 | 0.39 | 15.42 | 12.8 | 6.57 | 2.38 | 8 | 8 | 6 | 6 |
| 173 | survival | 28 | 30.94 | 24.48 | 23.05 | 35.91 | 8980 | 1950 | 648 | 633 | 100 | 100 | 48.1 | 13.1 | 16.29 | 12.06 | 3.01 | 2.59 | 12 | 11 | 9 | 4 |
| 174 | survival | 28 | 15.90 | 13.53 | 15.69 | 15.58 | 727 | 488 | 478 | 439 | 29.2 | 6.86 | 1.76 | 0.6 | 21.23 | 19.58 | 10.58 | 8.76 | 11 | 4 | 5 | 5 |
| 175 | survival | 28 | 18.64 | 20.77 | 24.13 | 20.16 | 11400 | 10800 | 7600 | 8230 | 35.6 | 9.91 | 3.6 | 4.3 | 24.43 | 6.85 | 4.07 | 5.21 | 16 | 7 | 5 | 6 |
| 176 | survival | 28 | 16.97 | 17.16 | 21.19 |  | 506 | 266 | 310 |  | 96.2 | 34.6 | 9.5 |  | 28.14 | 19.62 | 6.33 |  | 10 | 4 | 6 |  |
| 177 | survival | 28 | 14.78 | 11.07 | 12.52 | 14.83 | 1210 | 304 | 558 | 239 | 17.1 | 3.66 | 9.85 | 1.55 | 17.11 | 14.96 | 7.88 | 7.61 | 13 | 9 | 10 | 6 |
| 178 | survival | 28 | 13.05 | 12.09 | 11.21 | 13.77 | 1940 | 2040 | 2780 | 2870 | 35.5 | 10.1 | 3.62 | 1.06 | 15.7 | 21.42 | 6.41 | 3.19 | 16 | 12 | 9 | 8 |
| 179 | survival | 28 | 17.93 | 16.68 | 28.60 |  | 540 | 488 | 420 |  | 2.8 | 2.87 | 0.99 |  | 13.9 | 17 | 11.84 |  | 11 | 7 | 3 |  |
| 180 | survival | 28 | 9.98 | 8.94 | 16.63 |  | 850 | 517 | 986 |  | 66 | 5.56 | 0.9 |  | 11.56 | 38.72 | 9.87 |  | 12 | 10 | 5 |  |
| 181 | survival | 28 | 11.89 | 8.05 | 7.58 | 10.14 | 3190 | 2530 | 5980 | 2750 | 42 | 27.3 | 8.01 | 2.81 | 21.48 | 38.21 | 34.86 | 8.33 | 13 | 11 | 9 | 12 |
| 182 | survival | 28 | 7.82 | 10.37 |  |  | 1370 | 801 |  |  | 13.3 | 2.21 |  |  | 10.5 | 8.71 |  |  | 10 | 7 |  |  |
| 183 | survival | 28 | 20.62 | 24.37 |  |  | 878 | 815 |  |  | 79 | 16.7 |  |  | 33.3 | 15.3 |  |  | 9 | 7 |  |  |
| 184 | survival | 28 | 16.40 | 12.58 | 20.00 | 26.06 | 1230 | 1040 | 708 | 500 | 13.3 | 7.53 | 1.45 | 0.54 | 28 | 26.86 | 14.35 | 14.9 | 11 | 10 | 10 | 7 |
| 185 | survival | 28 | 20.52 | 23.38 | 32.20 |  | 460 | 323 | 243 |  | 7.19 | 2.07 | 1.21 |  | 28.5 | 15.7 | 9.2 |  | 13 | 8 | 1 |  |
| 186 | survival | 28 | 14.14 | 14.25 |  |  | 1950 | 540 |  |  | 78.2 | 29 |  |  | 32.7 | 17.2 |  |  | 10 | 5 |  |  |
| 187 | survival | 28 | 9.72 | 15.04 | 19.42 | 19.18 | 7750 | 6700 | 2180 | 1710 | 25.7 | 7.98 | 2.88 | 1.5 | 35 | 24.76 | 7.39 | 2.21 | 16 | 15 | 6 | 7 |
| 188 | survival | 28 | 13.15 | 16.96 | 16.36 | 16.26 | 1990 | 1420 | 814 | 675 | 10.7 | 4.52 | 1.4 | 0.7 | 26.8 | 15.4 | 7.41 | 0 | 5 | 3 | 3 |  |
| 189 | survival | 28 | 18.98 | 20.72 | 24.34 | 29.30 | 379 | 417 | 549 | 518 | 14.3 | 4.34 | 1.54 | 0.7 | 33.81 | 29.56 | 25.07 | 12.27 | 4 | 7 | 6 | 6 |
| 190 | death | 5 | 13.81 | 9.13 |  |  | 2590 | 3370 |  |  | 58.9 | 33.5 |  |  | 38.48 | 47.8 |  |  | 18 | 21 |  |  |
| 191 | survival | 28 | 18.05 | 25.13 |  |  | 1470 | 459 |  |  | 0.91 | 0.31 |  |  | 16.7 | 17.6 |  |  | 7 | 1 |  |  |
| 192 | survival | 28 | 25.23 | 29.47 |  |  | 3370 | 2340 |  |  | 5.29 | 1.2 |  |  | 21.3 | 21.98 |  |  | 9 | 5 |  |  |
| 193 | death | 2 | 6.15 |  |  |  | 5320 |  |  |  | 14.1 |  |  |  | 11.57 |  |  |  | 9 |  |  |  |
| 194 | survival | 28 | 27.12 | 17.27 |  |  | 243 | 305 |  |  | 49 | 13.4 |  |  | 8.97 | 19.89 |  |  | 9 | 8 |  |  |
| 195 | survival | 28 | 14.22 | 16.08 | 13.52 |  | 721 | 320 | 219 |  | 100 | 67.2 | 18.5 |  | 26.26 | 20.84 | 3.97 |  | 6 | 6 | 4 |  |
| 196 | survival | 28 | 14.06 | 16.20 | 20.27 |  | 1380 | 1010 | 541 |  | 5.23 | 1.42 | 0.39 |  | 15 | 5.8 | 1.83 |  | 5 | 5 | 2 |  |
| 197 | survival | 28 | 16.68 | 18.24 |  |  | 2320 | 1100 |  |  | 60.3 | 24.7 |  |  | 18.27 | 10.88 |  |  | 11 | 4 |  |  |
| 198 | survival | 28 | 23.13 | 21.18 |  |  | 3900 | 2420 |  |  | 2.46 | 0.64 |  |  | 31.3 | 19.04 | 6.02 |  | 13 | 8 | 3 |  |
| 199 | survival | 28 | 19.93 | 23.88 |  |  | 1210 | 389 |  |  | 18.9 | 5.19 |  |  | 19.71 | 13.86 |  |  | 9 | 3 |  |  |
| 200 | survival | 28 | 14.74 | 10.98 | 14.11 | 12.64 | 1050 | 2180 | 1390 | 987 | 84.3 | 48.7 | 16.7 | 15.8 | 15.56 | 26.33 | 9.7 | 7.46 | 9 | 13 | 7 | 6 |
